# Improved Mobilome Delineation in Fragmented Genomes

**DOI:** 10.3389/fbinf.2022.866850

**Published:** 2022-04-11

**Authors:** Catherine M. Mageeney, Gareth Trubl, Kelly P. Williams

**Affiliations:** ^1^ Systems Biology Department, Sandia National Laboratories, Livermore, CA, United States; ^2^ Physical and Life Sciences Directorate, Lawrence Livermore National Laboratory, Livermore, CA, United States

**Keywords:** metagenome-assembled genome mobile genetic element, genomic island, prophage, metagenomics, metagenomeassembled genome

## Abstract

The mobilome of a microbe, i.e., its set of mobile elements, has major effects on its ecology, and is important to delineate properly in each genome. This becomes more challenging for incomplete genomes, and even more so for metagenome-assembled genomes (MAGs), where misbinning of scaffolds and other losses can occur. Genomic islands (GIs), which integrate into the host chromosome, are a major component of the mobilome. Our GI-detection software TIGER, unique in its precise mapping of GI termini, was applied to 74,561 genomes from 2,473 microbial species, each species containing at least one MAG and one isolate genome. A species-normalized deficit of ∼1.6 GIs/genome was measured for MAGs relative to isolates. To test whether this undercount was due to the higher fragmentation of MAG genomes, TIGER was updated to enable detection of split GIs whose termini are on separate scaffolds or that wrap around the origin of a circular replicon. This doubled GI yields, and the new split GIs matched the quality of single-scaffold GIs, except that highly fragmented GIs may lack central portions. Cross-scaffold search is an important upgrade to GI detection as fragmented genomes increasingly dominate public databases. TIGER2 better captures MAG microdiversity, recovering niche-defining GIs and supporting microbiome research aims such as virus-host linking and ecological assessment.

## Introduction

The mobilome is the collection of mobile genetic elements (MGEs), such as transposable elements, plasmids, and prophages, present in a genome. Aside from selfish genes for propagation, an MGE can carry cargo genes that benefit the host organism, for example by promoting catabolism of organic pollutants (van der Meer and Sentchilo, 2003), nitrogen fixation (Sullivan and Ronson, 1998) or biofilm formation (Drenkard and Ausubel, 2002). Acquisition of a new cargo-bearing MGE can quickly and profoundly alter the phenotype of the host microbe. Therefore to understand the evolution and ecological role of microbes, it is important to delineate their mobilomes. If the genome is complete and closed, plasmids are automatically identified as isolated replicons, but precise identification of those MGEs that lie integrated within the chromosome is more challenging. The fragmentation accompanying incomplete genomes, typical of metagenome-assembled genomes (MAGs), further increases the challenge of identifying MGEs.

Genomic islands (GIs) are a subclass of MGEs that integrate into microbial chromosomes, usually with high specificity for a particular chromosomal site (*attB*), determined by the GI-encoded integrase. They range from ∼5 to hundreds of kbp and carry genes of diverse function. GIs can be horizontally transferred *via* conjugation, transformation or transduction, with mobility heavily influenced by other MGEs (Bertelli et al., 2019). Some GIs carry a gene set revealing the mode of transfer between microbes, either bearing conjugative genes that indicate an integrative and conjugative element (ICE), or viral genes that indicate a prophage, i.e., a temperate phage in the lysogenic phase of its life cycle. Other GIs are satellites, which do not carry their own transfer genes but require a helper, itself either an ICE or phage, to supply gene products promoting transfer (Fillol-Salom et al., 2018).

There are several computational GI prediction tools [reviewed in (Bertelli et al., 2019)] that exploit special GI features, such as sporadic occurrence within a species, differences from the nucleotide sequence composition of the chromosome, preference for tRNA genes, and gene content. Our methods Islander and TIGER are unique in their precise mapping of GIs (Hudson et al., 2015; Mageeney et al., 2020). Precise GI mapping improves genome annotation and allows discoveries of new *attB* site-specificity by integrases, site-promiscuous integrase clades, and cases where cells use GIs to regulate gene integrity.

The advent of metagenomics has reshaped our understanding of uncultured microbes and microbial communities. Early metagenomics provided mere gene catalogs of environmental samples, but the field has turned toward genome-centric characterization, as read-depth coverage and bioinformatic tools improved sufficiently to enable coverage-based binning of assembled scaffolds into population genomes or MAGs (Taş et al., 2021). Characterization of MAGs has revealed that high proportions of bacteria and archaea remain uncultured (Steen et al., 2019) and that most metagenomic reads do not map to any MAG or isolate genome (Nayfach et al., 2021).

MAGs are lower quality than same-species isolate genomes by every available metric (Supplementary Table S1). Some of the factors contributing to reduced MAG quality are similar to those that may plague any genome project: low coverage that can break or leave gaps in the assembly, and outright misassembly. The key feature distinguishing a metagenomic DNA sample from an isolate DNA sample is complexity. One way complexity manifests is through different levels of coverage for different microbes, exacerbating the low coverage problem for some MAGs in a metagenome. Complexity can also manifest as microdiversity, where a group of population-level variants exist in the sample. Resolution of multiple individual MAGs from the same microdiverse population is often impossible but has been achieved when species diversity is low (Tyson et al., 2004) or complexity is reduced (Sieradzki et al., 2020; Haro-Moreno et al., 2021; Nicolas et al., 2021). More often a single consensus MAG can be obtained for a population with moderate microdiversity, but high microdiversity can counteract assembly, perhaps leaving the more diverse genomic regions unassembled and reducing the completeness of the MAG. Finally, a problem unique to metagenomes can occur post-assembly, at the binning step (Evans and Denef, 2020). Shared nucleotide sequence composition of scaffolds is a major basis for binning, such that genomic regions departing from baseline composition can be misbinned, generating artifactual composite MAGs (Shaiber and Eren, 2019). We have observed cross-domain misbinning, where scaffolds with uniquely bacterial markers are mixed into archaeal MAGs (unpublished results).

There has been relatively little emphasis in the literature on the problems that metagenomic datasets pose for mobilome delineation. Scaffolds from within MGEs are more prone to misbinning because they can strongly differ in composition from their surrounding chromosomes (Carr et al., 2020; Maguire et al., 2020). MGEs tend to have higher microdiversity than chromosomal regions because MGE gene expression is largely repressed, reducing selective pressure to preserve MGE nucleotide sequence (Haro-Moreno et al., 2021). Finally, induction of a GI, i.e., its excision, circularization and possible replication in some cells within a MAG population, can confuse assemblers. We have observed such assembler confusion caused by inadvertent GI induction in isolate assemblies (unpublished results). Alternative GIs at the same genomic site is another formal possibility for a type of diversity that could affect assembly of MAGs. MGEs are not included in the assessment of MAG quality (Bowers et al., 2017); a MAG may thus be considered high quality, yet still be missing extensive portions of its mobilome.

**FIGURE 1:**
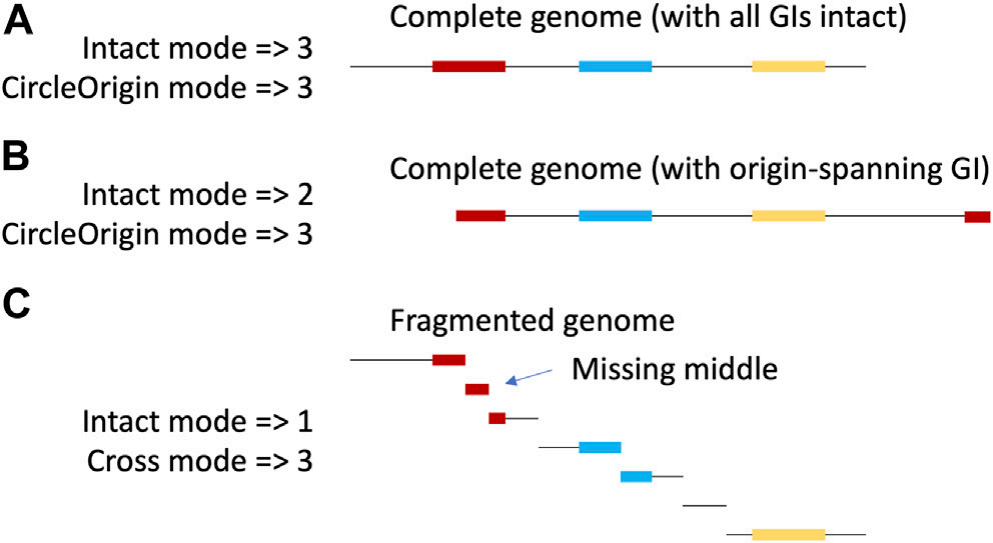
New TIGER modes. The same circular chromosome with 3 (colored) GIs is shown with a complete **(A,B)** or fragmented assembly **(C)**. With complete assembly, if the origin of the linearized sequence of the circle is randomly chosen, it will occasionally fall within a GI, splitting the GI **(B)**. Yields are shown for the various TIGER modes. The original mode can only find intact GIs on a single scaffold, while the new modes, CircleOrigin (applied to complete assemblies) and Cross (applied to fragmented assemblies), can additionally find the split islands. Because TIGER focuses on GI-flanking sequences, the Cross-mode call for a multiply split GI (red in panel C) will only include the terminal fragments and exclude middle GI fragments.

Here, we present TIGER2, with new modes to identify GIs either across two contigs or around the circular origin of a chromosome (Figure 1), doubling average GI yields.

## Materials and Methods

Genomes. We collected a set of 74,561 genomes (for 7978 MAGS and 66,583 isolates) from 2,473 microbial (64 archaeal, 2,409 bacterial) species, where each species contained at least one MAG and one isolate genome (Supplementary Table S2). We downloaded 288,451 microbial genomes from GenBank in July 2019, after rejecting additional genomes with N50 < 10,000 or scaffold count >300. A script speciate. pl was developed employing MASH and fastANI that placed all but 1,656 of the GenBank genomes into a species defined by GTDB release 202 (Parks et al., 2022); for the 173,660 GenBank assembly IDs that had been treated by GTDB, which applies its own genome quality filters, the script mismatched the GTDB assignment in only 184 rejected cases, at least some due to major differences between versions of the assemblies. Among the 47,894 GTDB species, 2,487 were found to contain at least one MAG and one isolate genome. All remaining MAG genomes for these species, and many remaining isolate genomes (up to 200 total per species unless more were already available) were collected. Fourteen two-genome species were rejected in which the two genomes had identical scaffold size lists, suggesting duplicate entries.

TIGER version 2. TIGER was originally designed to map intact GIs present on a single scaffold. We re-wrote the core software to offer two new “split” modes that yield split GIs, in addition to the intact GIs (Figure 1). “CircleOrigin” mode finds split GIs that wrap around the origin of a circular replicon. “Cross” mode detects split GIs with termini on separate scaffolds. We applied CircleOrigin mode to the 9 008 genomes we considered complete (in five or fewer parts, to accommodate plasmids and secondary chromosomes), and applied Cross mode to the 65,553 remaining, fragmented genomes. To accommodate the new split GIs, the main TIGER wrapper and the merge. pl script that produces a tentative file of nonoverlapping GI calls were also revised, but we have not yet revised the orthogonal software Islander nor the resolve. pl script that compares Islander/TIGER calls and treats tandem GI arrays. New software is available at github/sandialabs/TIGER.

Genomic islands. TIGER is a comparative method, requiring a database of reference genomes. We prepared a tailored database for each species consisting of all genomes for that species, capping at 200. For species with ≥ 200 genomes, the most diverse 200 were chosen based on all vs. all MASH distance scores. TIGER2 was run in Intact and either Cross or CircleOrigin modes on all genomes through to the merge. pl script, and GIs were collected from the resulting genome. island.nonoverlap.gff files above a size cutoff of 5 kbp, containing a serine (S-Int) or tyrosine (Y-Int) integrase gene, and with crossover length <300 bp, allowing overlaps no larger than 100 bp. This yielded 223,323 GIs identified by both modes, 211,599 identified by split-scaffold mode only and 13,653 identified by same-scaffold mode only.

Typing of split GIs. TIGER typing software was adapted to handle split GIs. The two halves of the split GI are annotated with our Tater software (Mageeney et al., 2020) which uses Prodigal to call open reading frames, Prokka to assign gene names, and applies Pfam-A HMMs (v. 35) including subsets for phage and ICE proteins. Typing proceeds according to gene content of the entire split GI, as previously described (Mageeney et al., 2020). This yields seven output categories: Phage1, GI containing at least one structural and at least one non-structural phage Pfam; Phage2, GI containing at least one phage Pfam; PhageFil, GI less than 13 kb that contain the Pfam Zot, previously identified in many Inoviridae phages (Roux et al., 2019); ICE1, GI with ≥7 or ≥15% ICE Pfams; ICE2, GI under 10 kb with >2 or ≥12% ICE genes, or over 10 kb with >2 or >7% ICE genes; PhageICE, GI matching both Phage and ICE criteria (very rare and usually due to mistaken grouping of neighbors in a tandem array); Other, GI with none of the above calls.

Testing large groups of islands. Four GI-abundant genomic loci, the *Escherichia icd*, tmRNA, and *ybhC/ybhB* loci and the *Mycobacterium* tRNA-Ser locus, were studied to examine the quality of the split GI calls. GI sequences were collected for the intact and split islands assigned to those sites, and the 600 bp *attL* and *attR* terminal GI-internal segments were taken as queries, except in cases where scaffold splitting left the terminal segment shorter than 600 bp, where the segment contained a transposase gene indicating sequence likely to be repeated throughout the genome, or where long blocks of ambiguous bases precluded even self-matching. Strong matches (≥500 bp and ≥95% identity) in all-vs. all BLASTN of the intact GI termini were clustered as connected components, combining *attL* and *attR* typing to produce the *attP* type for each intact GI.

## Results

GI yields for MAGs and isolates. As a null hypothesis, MAGs could be expected to contain numbers of GIs comparable to isolate genomes. Because some phylogenetic groups are more GI-rich than others (Mageeney et al., 2020), we reasoned that MAG/isolate comparisons would be most appropriate within a species, and that large numbers of such within-species comparisons could achieve statistical significance. The GTDB project has systematically treated most archaeal and bacterial genomes, applying a revised taxonomy that we employ here to improve genome comparison (Parks et al., 2022). Its most strictly defined rank is the species; each is seeded by a representative genome, and a genome must have 95–97% similarity to the representative for inclusion in the species. We analyzed 2 473 GTDB species containing at least one MAG and one isolate genome, totalling 74,561 genomes (7,978 MAGs and 66,583 isolates). Despite this overall bias toward isolates, 894 species had equal numbers of MAGs and isolates, and 549 had more MAGs than isolates.

**FIGURE 2:**
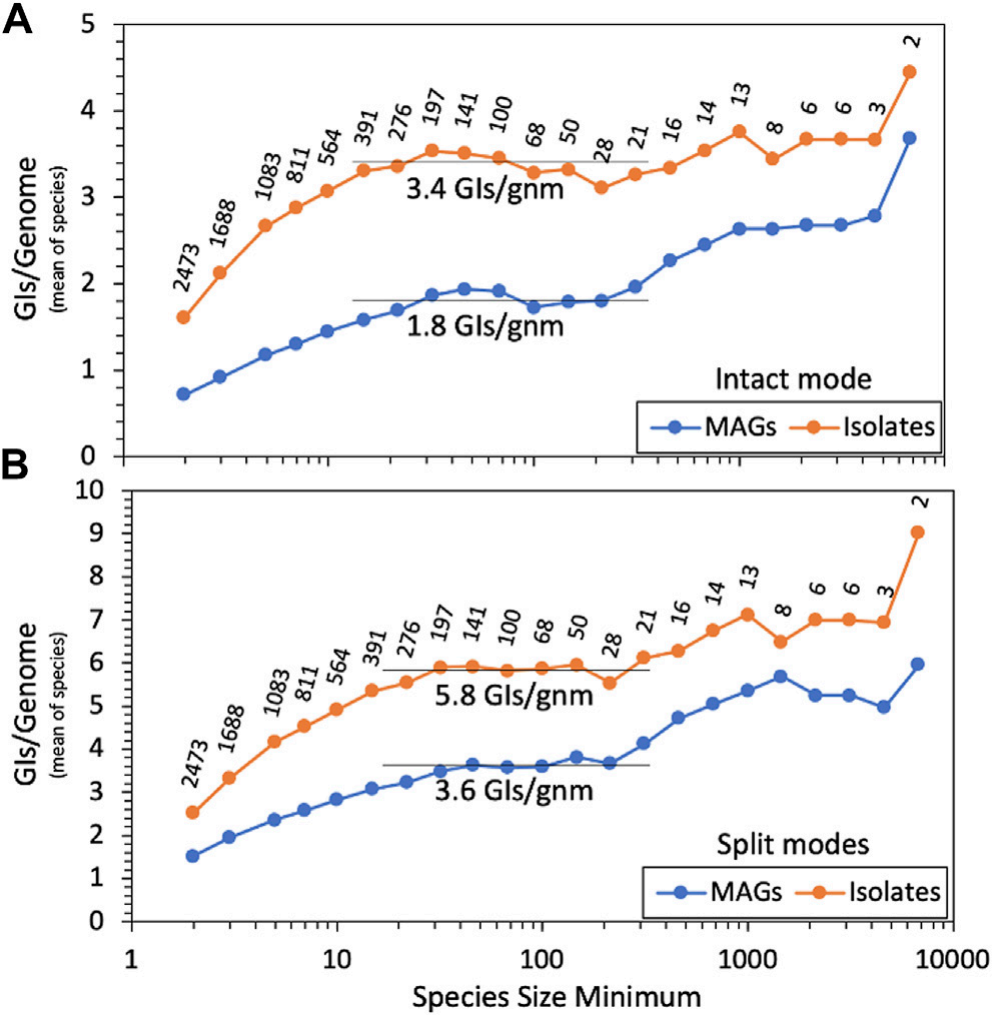
GI yields for MAGs and isolate genomes. TIGER2 was run in **(A)** intact-only mode or **(B)** split modes on genomes from 2473 GTDB species containing at least one MAG and one isolate genome, measuring GIs/genome within each species; shown here is the mean of the GI/genome values for all species tested at each size (i.e., genome count) cutoff. Data labels show the numbers of species remaining with each size cutoff.

We ran our GI discovery software TIGER on these genomes, counting GI yields for each. Average GI recovery over all MAGs or isolates would be misleading and dominated by relatively few overrepresented species due to the wide range of species sizes, from 2 to 9 114 genomes. MAG and isolate GI yields were averaged within each species, and we present (Figure 2A) averages over all species, using various cutoffs for species size. For both small and large species, there is a trend of increased GI yields with increasing species size. At the left of the figure, small species had small reference databases for TIGER, which likely explains their lower yields. The right of the figure suffers from noise due to low species numbers. The middle region is flatter and provides a species-normalized estimate of 3.4 GIs per isolate genome, with a large depression for MAGs, down to 1.8 GIs per genome. This depression is probably explained by the poorer quality of MAG genomes, worse than isolates by every available metric (Supplementary Table S1). Especially relevant is scaffold counts, averaging 95 for isolates and 152 for MAGs. TIGER was designed to search for GIs contained within a single scaffold, but in fragmented genomes, some GIs may also be fragmented, escaping detection.

TIGER2. TIGER employs a “ping-pong BLAST” method, first running a query sequence from the study genome (a candidate GI/chromosome boundary proximal to an integrase gene) against a reference genome database, then running a second query from each hit reference genome back to the original scaffold of the study genome, to find the distal end of the intact GI. In principle this second query can be applied to *all* scaffolds in the study genome to find GIs split among contigs. TIGER2 allows the original “Intact” mode that only finds within-scaffold GIs and two new split modes (either “Cross” for fragmented genomes or “CircleOrigin” for complete genomes) that can also find the termini of GIs when split onto different scaffold ends. We also prepared new species-focused reference databases (Materials and Methods, “Genomic islands”). Running the split modes on the genomes produced many more GI calls. There were 223,323 GIs for which intact and split modes agreed, 13,653 found by intact mode only, and a surprisingly large number, 211,599, found by split modes only. All GI calls from TIGER2 are reported in Supplementary Table S2. Repeating the yield analysis (Figure 2B), the split modes improved GI yields 1.7-fold for isolates and 2.0-fold for MAGs, elevating the MAGs:isolates ratio from 0.52 (intact mode) to 0.62 (split modes).

The split GIs are generally better supported than competing intact GI calls. A support value is computed for each GI call equal to the number of reference database genomes found to be precisely deleted for (and thereby mapping) the GI. For the 11,152 contests where a split-only GI overlapped an intact-only GI, 806 were tied for support, 543 of the contests were won by higher support for the intact-only GI, and 9,419 were won by the split-only GI.

Assessing GIs at four common genomic integration sites. To further assess the quality of TIGER2 calls, four large groups of GIs integrating into the same genomic site in the same large genus were examined, at the *icd* and tmRNA genes and the phage lambda locus (the *ybhC/ybhB* intergenic site) of *Escherichia*, and the tRNA-Ser gene in *Mycobacterium* (Table 1). The *Mycobacterium* tRNA-Ser gene (and other loci in the genus) have far fewer split-only GIs than the *Escherichia* loci. This may be simply explained by the much larger scaffold:GI length ratio, 11.0, for tRNA-Ser GIs in *Mycobacterium*; this ratio is only 1.6–1.8 for the *Escherichia* GIs. Databases were prepared from the genomes containing an intact GI at the site for the genus. For each locus, the GI-internal terminal DNA sequences were used to type intact and split GIs, and a split GI was considered validated when its termini matched those of an intact GI. This test of GI-*internal* sequences is orthogonal to the TIGER method itself, which finds GIs based only on their *flanking* sequences. The sequences from the *attL* region were independently typed, as were the *attR* sequences, and together these produced an *attP* type for each GI. Although the goal of this typing was to assess the new split GIs, we first characterized *attP* types among intact GIs only.

**TABLE 1:** Validation of split GI calls at four commonly used integration loci. Analysis of the tRNA-Ser locus was from 6,283 *Mycobacterium* genomes and of the *icd*, tmRNA and lambda loci from 15,111 *Escherichia* genomes.

Locus	Icd	tmRNA	Lambda	tRNA-ser
Total GIs	3,905	4,882	4,651	6,155
Found by intact and split modes	1,379	2,246	1,248	6,088
Found by intact mode only	10	53	0	6
Found by split modes only	2,516	2,583	3,403	61
Split mode, novel intact	3	4	0	11
Circular origin spanning	7	2	4	0
Cross-scaffold	2,506	2,577	3,399	50
Intact GIs typed	1,361	2,193	1,110	6,099
Intact GI *attL* types	31	100	137	7
Intact GI *attR* types	29	154	8	7
Intact GI *attP* types	66	278	140	7
Split GIs typed	2,361	2,357	2,856	46
Split GIs, known *attP* type	2,286	2075	2,726	46
Split GIs, novel *attP* type	75	282	130	0

Intact GIs at the four integration sites. At the *Mycobacterium* tRNA-Ser locus, only seven *attP* types were observed, that do not mix *attL* and *attR* types, and are strictly segregated by species, for example, the largest type (6,075 GIs) is restricted to *M. tuberculosis* (Mtu) and is the only *attP* type in that species (Table 1). At the *Escherichia* loci there is much greater *attP* type diversity, strong but imperfect species segregation, and each shows mixing of the half-*attP*s. For example, between two abundant *attP* types at *icd*, L1-R2 (this designation indicates its composition from *attL* type 1 and *attR* type 2) and L10-R4, both mixtures are observed, L1-R4 and L10-R2. Such swapping of unrelated *attP* halves is probably an example of the mosaicism that is pervasive among GIs, but in some cases could be due to unresolved tandem GI arrays. At the lambda site, we observe lopsided mosaicism: one main *attR* type and many different *attL* types. The tmRNA gene has the highest occupancy and the highest diversity of *attP* types, perhaps related to its known targeting by multiple independent integrase clades (Williams, 2003).

The Mtu tRNA-Ser GI reveals a problem with using small, single-species reference databases; this GI is so widespread in the species that only one of the 200 genomes in the Mtu reference database was lacking the GI and therefore able to identify, map, and support it. With a support value of only one, a false positive GI call with support values as low as two might overlap the tRNA-Ser GI and eliminate it during the merging step. Six false negative intact Mtu GIs were identified through matches to the split GI queries; all had been identified by the TIGER core module, but rejected during merging due to overlapping false positives. In the future we will prepare reference databases that include some genomes from outside the species.

Split GIs at the four loci. Results for the tRNA-Ser locus can be succinctly summarized. The 31 split GIs from Mtu all had the same *attP* type as all intact Mtu GIs. The remaining 15 split GIs, from *M. immunogenum*, had an *attP* type of intact *M. immunogenum* GIs. For the *Escherichia* loci there were more split GIs than intact GIs, and some new *attP* types. Altogether 93.6% of the tested split GIs were validated, matching *attP* types known from intact GIs. Some of the mismatches may reflect additional mosaicism (Table 1).

**FIGURE 3:**
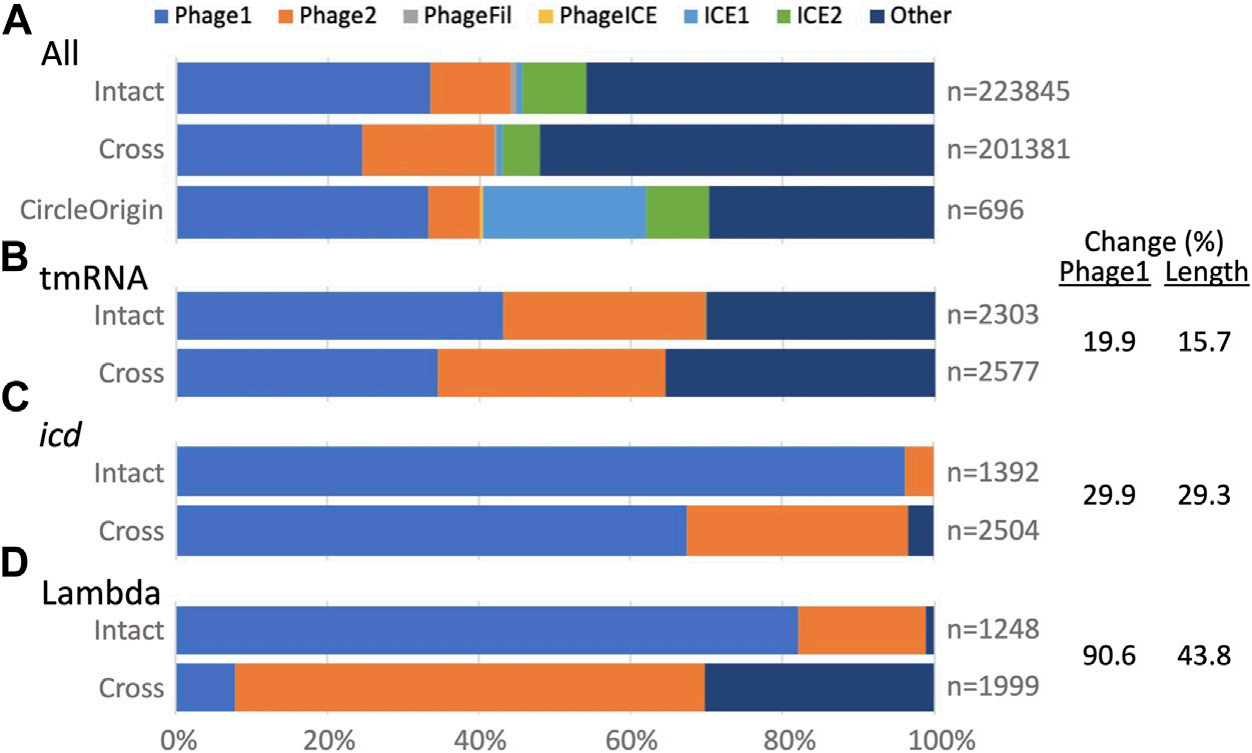
TIGER2 GI type breakdowns for composition categories (Intact, Cross and CircleOrigin). **(A)** All GIs, or **(B–D)** GIs at three *Escherichia* loci. Percent change is given for Intact vs. Cross GIs; change for Phage1 counts correlates with change in GI length across the three loci.

GI typing. The TIGER typing module determines whether a GI is a credibly complete prophage (Phage1) or contains less than a full complement of phage genes (Phage2), and likewise assigns a category one and two for ICEs, otherwise leaving the type undetermined (Other). This module was updated to accommodate split GIs. Examining all GIs (Figure 3A), the type breakdown for intact GIs is similar to that observed before (Mageeney et al., 2020), with almost half labeled Other and the next largest fraction labeled Phage1. For cross-scaffold GIs, the Phage1 fraction is appreciably smaller, while Phage2 and Other fractions are larger than for intact GIs. This “downward” typing shift may be due to “missing middles,” that is, if a GI is split onto more than two scaffolds, its central fragments would remain unidentified because TIGER2 finds only the terminal fragments of split GIs (Figure 1). Circle Origin GIs, which should not suffer from missing middles, have the same fraction of Phage1 as the intact GIs, with a notable expansion of the ICE1 category. ICEs tend to range to larger sizes than prophages; the arbitrary origin point of complete circular chromosomes may land more frequently on these larger GIs.

We also examined typing for GIs at the above three *Escherichia* sites, which all had large numbers of both intact and split GIs. For intact GIs, each site showed a different balance between Phage1, Phage2 and Other calls (Figures 3B–D). All had a downward typing shift for split GIs. According to our “missing middle” hypothesis, this downward typing shift might correlate with shorter split GI calls that omit central fragments. Extents of downward typing did indeed correlate with reductions in GI length (Figures 3B–D). For the split GIs at the tmRNA gene, the drops in Phage1 type and average GI lengths were small (20 and 16%). At the other extreme, the Phage1 fraction for the lambda site GIs dropped by 91% and the average GI length concomitantly dropped by 44%. Some features in many lambda site GIs may especially antagonize assembly, leaving more missing middle segments than for the tmRNA and *icd* GIs.

## Discussion

Our original GI detection software, operating only on single scaffolds, yielded substantially fewer GIs for MAGs than for species-matched isolate genomes. Suspicion that this was due to higher fragmentation of MAGs than isolates motivated a software update enabling cross-scaffold search. TIGER2 doubled GI yields for MAGs. This surprisingly large improvement shows that fragmentation levels in current microbial genomes substantially impact GI detection. Even with this new approach, MAG yields are still not equal to same-species isolate yields. A possible biological reason for this remaining discrepancy might be sought in the “domestication” of isolates through many generations of passage in the lab (Barreto et al., 2020); however we expect the opposite trend from domestication, that GIs could only be lost by excision events in isolates. Other aspects of quality such as completeness may depress yields in MAGs, when high microdiversity within a GI prevents its full assembly into a scaffold (Haro-Moreno et al., 2021). A third explanation is that only very small databases of related genomes may be available for many MAG-rich species, insufficient for TIGER (or any comparative method) to find all GIs.

The quality of the new split GIs is high by several criteria. GI support values outscore those of competing calls by intact-mode TIGER. At frequently-used genomic loci of integration, the split GIs share the *attP* compositions of the intact GIs. Split GIs have type profiles (phage:non-phage) comparable to intact GIs, although with a shift downward explainable by missing middle segments; TIGER2 finds only the terminal fragments of a GI such that its call will omit any additional internal fragments that might exist for the GI.

## Conclusion

Cross-scaffold search by TIGER2 doubles GI yields across diverse microbial species, linking more scaffolds and improving the quality of fragmented genomes such as MAGs. This will aid detection of viruses in metagenomic datasets, offer insights into population microdiversity and its phenotypic and ecological consequences, and help address questions such as the balance of temperate phages between the lysogenic state and free virions. We will apply TIGER2 to our larger genome database to produce an atlas of MGEs with precisely mapped termini in microbial genomes; although applied here only to GIs, the TIGER principle also discovers and maps other MGE classes, such as transposable elements (Mageeney et al., 2020). The rise of long-read sequencing is a welcome trend that will improve mobilome representation in MAGs; lengths can now be attained sufficient to contain an entire GI within a single read (Warwick-Dugdale et al., 2019; Nicolas et al., 2021; Zablocki et al., 2021).

## Data Availability

The datasets presented in this study can be found in online repositories. The names of the repository/repositories and accession number(s) can be found in the article/Supplementary Material.
